# Supplementary material for: Ectopic cervical thymi and no thymic involution until midlife in naked mole rats
Source: Aging Cell. 2021 Oct 1;20(10):e13477. doi: 10.1111/acel.13477 (PMC8520710; doi:10.1111/acel.13477)
Supplement: Supplementary file 1 — Figures S1‐S4 [file ACEL-20-e13477-s002.pdf]

Supplementary Figures S1-S4

Supplementary Figure 1

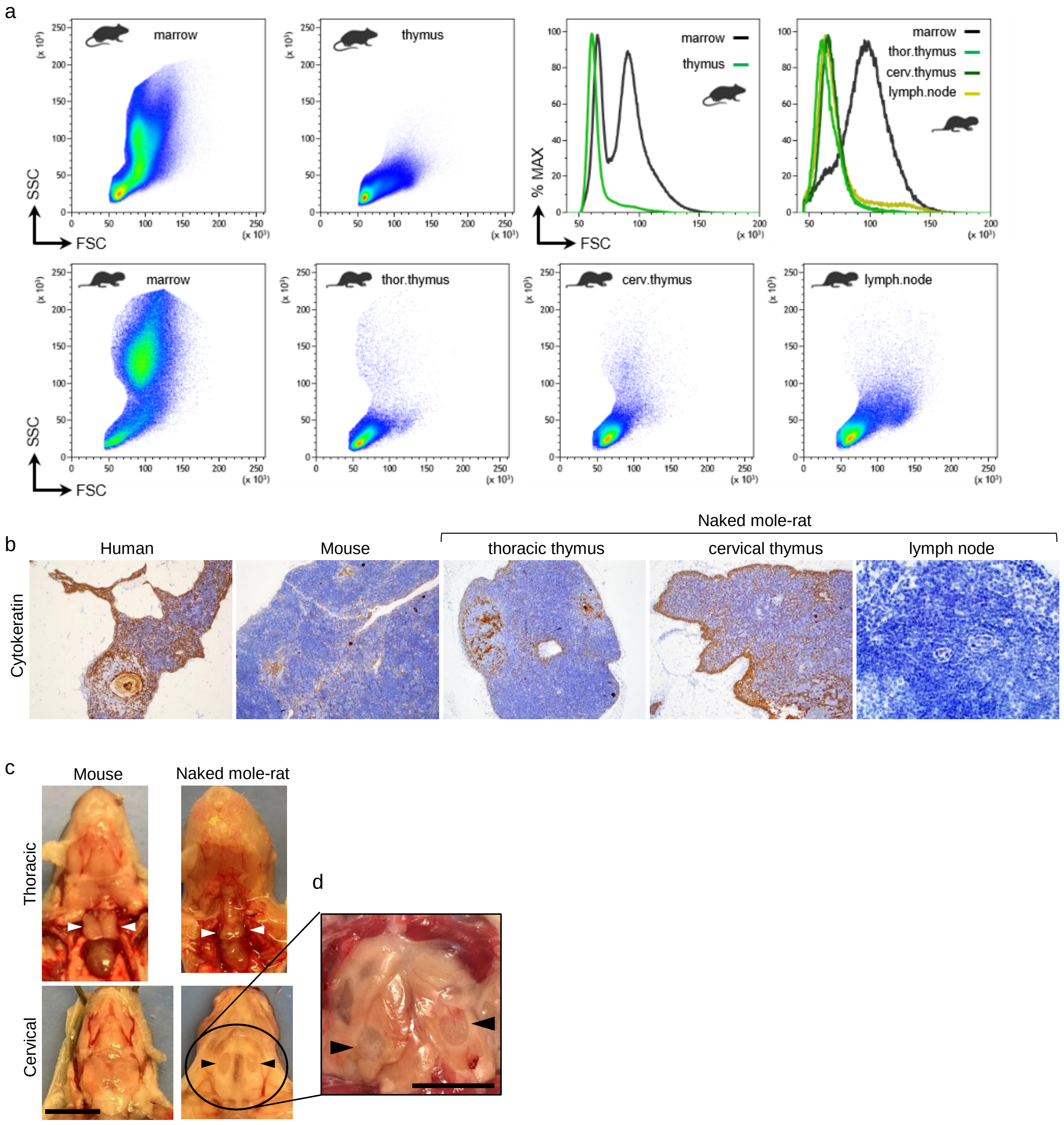

Figure S1. Flow cytometry and histology of mouse and naked mole-rat thymi.

a, Viable white blood cells (WBCs) of mouse marrow or thymus were compared for cell size using forward scatter (FSC) intensities, showing mouse thymocytes with cell sizes close to marrow lymphocytes. Same can be seen for naked mole-rat thoracic or cervical thymocytes or lymph node lymphocytes, showing same size as marrow lymphocytes. **b**, Cytokeratin staining of indicated thymus tissues; scale bar 200µm. **c**, Micrographs from the thoracic or cervical region of mouse and naked mole-rat neonates; scale bar 0.5cm. A bilobular thoracic thymus (white arrowheads) is present in both species, albeit drastically reduced in size in naked mole-rats. The cervical thymus (black arrowheads) is absent in mice, whereas in naked mole-rats the lobe is slightly larger on the neonate's right side, indicating a similar transient left-right asymmetry in developmental timing and morphology as seen for murine thymic organogenesis (Gordon & Manley,2011). **d**, Micrograph from the cervical region of an adult naked mole-rat (2.7 year-old); scale bar 0.5cm.

Supplementary Figure 2

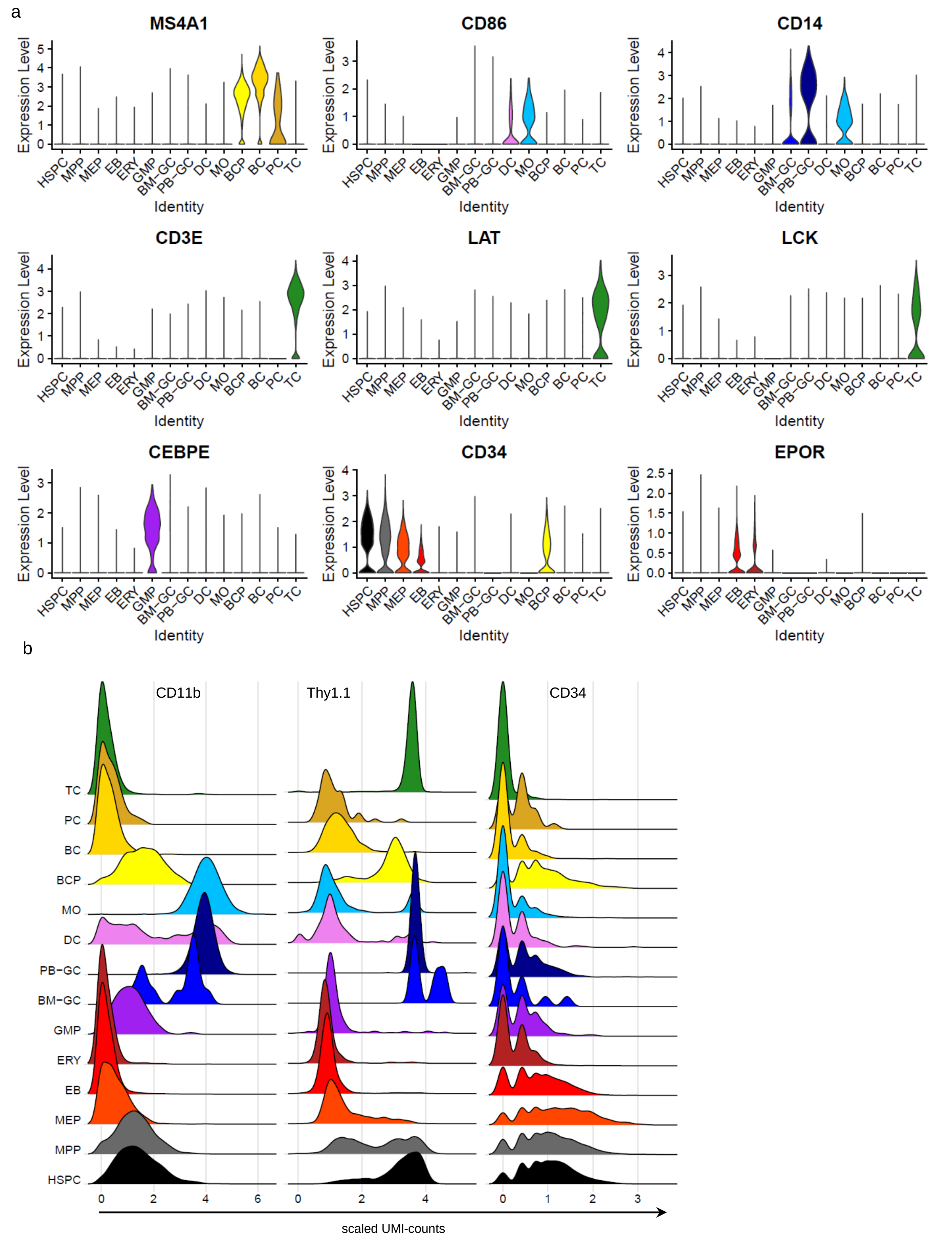

Figure S2. Naked mole-rat marrow and blood cell types derived from CITE-Seq.

a, Scaled RNA-UMI counts for CD20 (MS4A1), CD86, CD14, CD3E, LAT, LCK, CEBPE, CD34 and EPOR. HSPC, hematopoietic stem and progenitor; MPP, multipotent progenitor; MEP, megakaryocytic erythroid progenitor; EB, erythroblast; ERY, erythroid cells; GMP, granulocytic monocytic progenitor; BM-GC, marrow neutrophils; PB-GC, blood neutrophils; DC, dendritic cells; MO, monocytes; BCP, B cell progenitor; BC, B cells; PC, plasma cells; TC, T cells. **b**, CITE-UMI counts for indicated cross-reactive antibodies. Naked mole-rat TCs are CITE-CD11b^{-}/CD34^{-} /Thy1.1^{+}. Dataset derived from (Emmrich et al., 2021).

Supplementary Figure 3

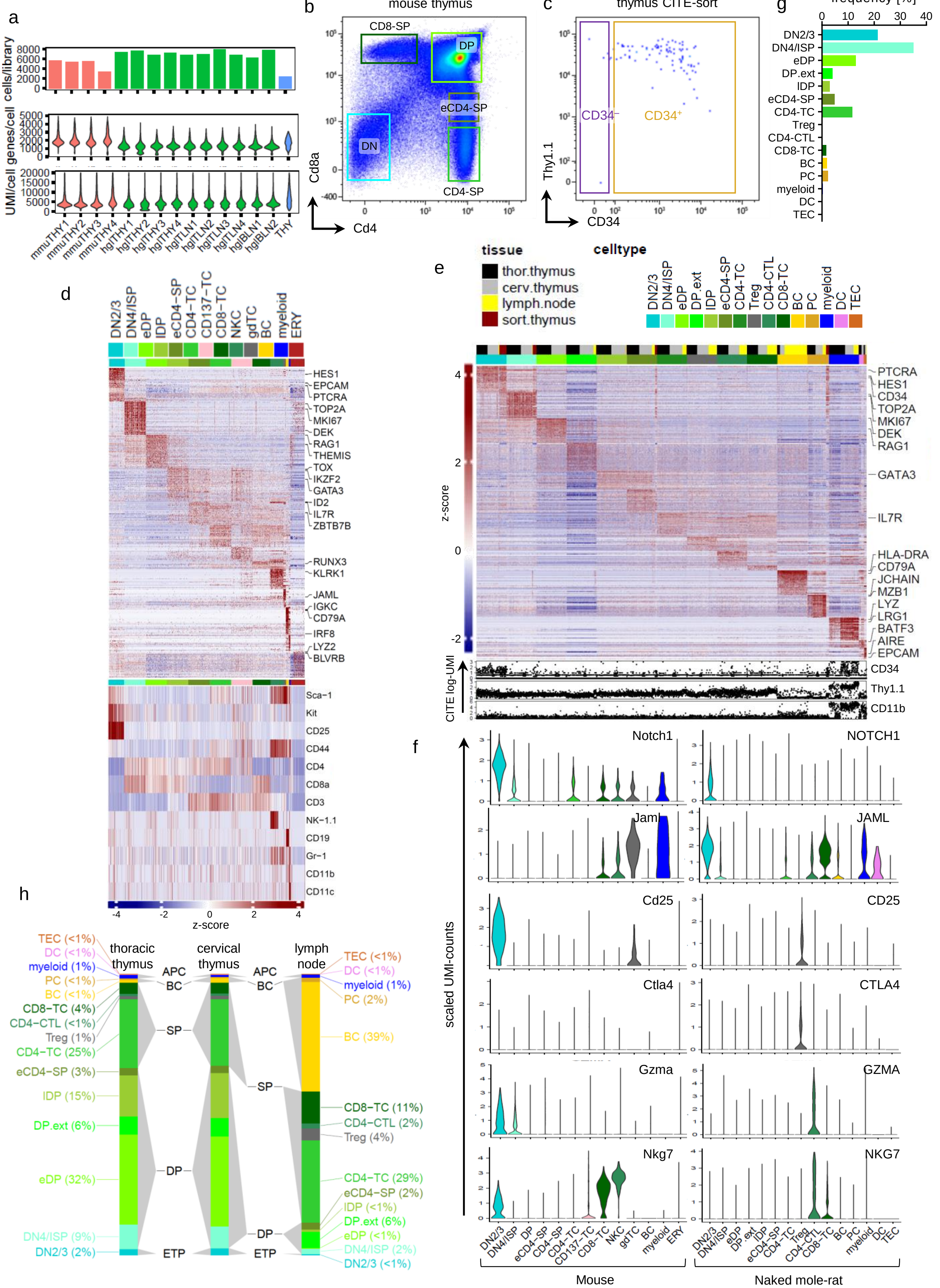

Figure S3. Single-cell RNA-Sequencing of mouse and naked mole-rat thymi.

a, Quality metrics for thymus scRNA-Seq datasets from Figure 6. Top, detected genes per cell; Bottom, mRNA UMI counts per cell. THY, thoracic thymus, TLN, cervical thymus, BLN, lymph node; *mmu*, mouse; *hgl*, naked mole-rat. **b**, FACS gating of Cd4 vs Cd8a on thymocytes from a 3m old mouse. DP, $Cd4^+/Cd8a^+$ double-positive; CD8-SP, $Cd4^–/Cd8a^+$ single-positive; eCD4-SP, $Cd4^+/Cd8a^{lo}$ early single-positive; CD4-SP, $Cd4^+/Cd8a^–$ single-positive; $Cd4^–/Cd8a^–$ double-negative. T-lineage specification onsets with TSPs through a series of well-characterized developmental checkpoints (Schlenner & Rodewald,2010). Termed double-negative (DN) these cells are $CD4^–/CD8^–$ and further subdivided by the expression of CD44 and CD25. Heterogeneous $CD44^+/CD25^–$ DN1 cells have the potential to form αβTC, γδTC, NKC, DCs, MAC and BC. $CD44^+/CD25^+$ DN2 cells start TCR-β, TCR-γ and TCR-δ gene segment rearrangements, DN3 cells ($CD44^–/CD25^+$) with successfully rearranged TCR-β chain enforce β-selection by association of an invariant pre-TCR-α chain (PTCRA) with CD3 signaling molecules to form the pre-TCR complex (Shah & Zuniga-Pflucker,2014). **c**, Postsort quality control with 102 viable events of the sorted naked mole-rat $CD34^+$ thymus sample, gating refers to sort decision. **d**, Top heatmap shows the top 25 cell type specific mRNA markers for mouse thymus randomly downsampled to ≤ 500 cells, canonical cell type markers from the literature are indicated. Bottom heatmap shows the cell type specific CITE features for mouse thymus randomly downsampled to ≤ 100 cells. Fold-change cut-off 2, p-value threshold 0.05. The CD137-TC cluster is partially composed of CITE-$CD4^–$ cells, while TNFRSF9 (CD137) mRNA was 5.3-fold upregulated (expressed in 45.7% CD137-TC vs 1.1% in all other clusters combined; adjusted $p<10^{-304}$). **e**, Heatmap of the top 25 cell type specific mRNA markers for the naked mole-rat lymphoid dataset from Figure 6E randomly downsampled to ≤ 500 cells, canonical cell type markers from the literature are indicated. Bottom traces depict differentially expressed CITE features as log-UMI counts. Top color bar labels tissue origin (see legend bottom right), 2nd color bar labels cell type annotation.

Fold-change cut-off 2, p-value threshold 0.05. **f**, Violin plots of gene expression across clusters for the mouse and naked mole-rat dataset. **g**, Cell frequencies of each cluster obtained from naked mole-rat sorted CD34+ thymocytes. **h**, Average cluster frequencies across unfractionated tissues; thoracic and cervical thymus vs LN. ETP, early T progenitor; DP, $CD4^+/CD8^+$ double positive SP, single-positive; BC, B cell; APC, antigen-presenting cell.

Supplementary Figure 4

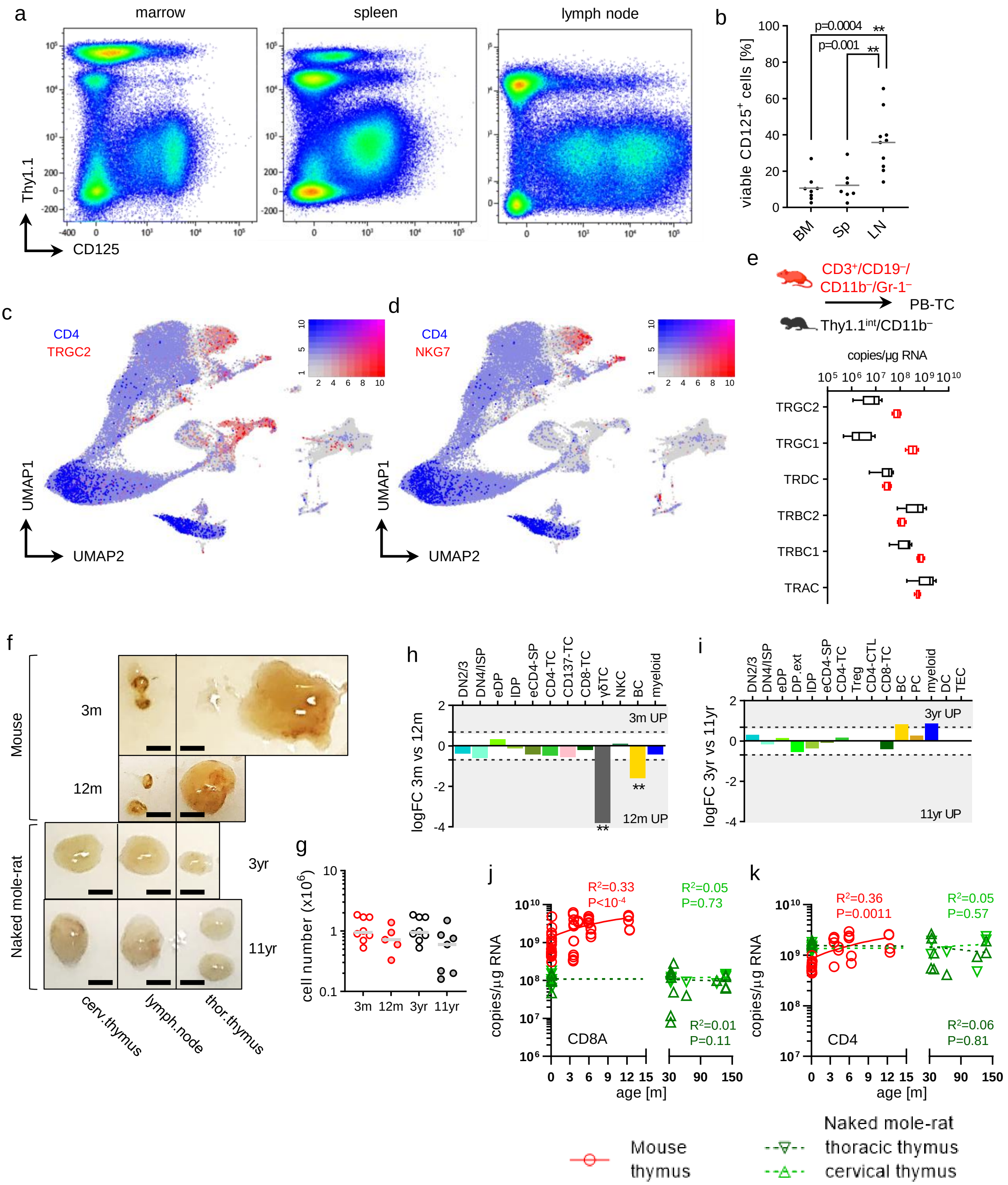

Figure S4. ScRNA-Seq continued and qPCR from mouse and naked mole-rat thymi.

a, FACS gating of $CD125^{+}$ BCs in naked mole-rat BM, spleen and lymph node. Note that the lymph node $CD125^{+}$ population has two separate $CD125^{dim}$ and $CD125^{bright}$ fractions. This was seen in 1/5 animals with no apparent disease condition noticed elsewise, other animal lymph nodes showed $CD125^{+}$ staining pattern comparable to BM. **b**, Frequencies of $CD125^{+}$ in BM (n=8), Spleen (n=7) and lymph nodes (LN, n=10); p-value determined by Dunnett's One-way ANOVA. **c**-**d**, UMAP-based Blendplots showing pairs conserved lineage markers; gene1 (red, high expression), gene2 (blue, high expression) and co-expressing cells (purple). See scale on the right; expression, scaled UMI counts. Cell type markers: CD4 3.7-fold up in eDP, 2.6-fold up in lDP, 2.7-fold down in CD4-CTL, 2.9-fold down in eCD4-SP and TEC, 3.1-fold down in DN4/ISP, 3.5-fold down in myeloid, 5.3-fold down in DN2/3, 5.9-fold down in PC, 6.1-fold down in CD8-TC, 6.6-fold down in BC; TRGC2 11-fold up in DN2/3, 6-fold up in CD4-CTL, 2.5-fold up in CD8-TC, 2.3-fold up in Treg; NKG7 14-fold up in CD4-CTL, 3.2-fold up in CD8-TC. **e**, Absolute qPCR of sorted T cells from mouse (n=4) and naked mole-rat (n=5) for T cell receptor constant chain orthologs; p-value derived from Tukey's Two-way ANOVA. **f**, Micrographs of perfused thymi and lymph nodes from mice and naked mole-rats, each age group represents one photograph of aligned tissues from the same animal. All photographs included a ruler next to the tissues, by which images were adjusted to a common size, represented by the scale bar 2mm. Nodes for all animals extracted from cervical/pharyngeal region. The rather puzzling finding of same-sized cervical LNs with 10-fold less cell content than cervical thymi could be explained by a one-size-fits-all encapsulation within HMW-HA, which is abundantly found across most major tissue types in naked mole-rats (Tian et al., 2013b). **g**, Cellularity of 3m (n=8) and 12m (n=5) old mice vs 3yr (n=8) and 11yr (n=7) old naked mole-rat cervical lymph nodes; p-value derived from Sidak's One-way ANOVA. Differential cell type abundance across age for **h**, mouse or **i**, naked mole-rat single-species analysis. *mmu* BC, p=0.006; *mmu* γδTC, p=0.011. Absolute copy number determination

for **j**, CD8A or **k**, CD4 ortholog mRNA in whole mouse (n=28) and naked mole-rat thoracic (n=12) and cervical (n=15) thymi. R^2 and p-value derived from linear regression; Legend below used for both panels.
